# Protective Effect of Resveratrol on Knee Osteoarthritis and its Molecular Mechanisms: A Recent Review in Preclinical and Clinical Trials

**DOI:** 10.3389/fphar.2022.921003

**Published:** 2022-07-25

**Authors:** Shenglei Yang, Mingli Sun, Xinan Zhang

**Affiliations:** School of Kinesiology, Shenyang Sport University, Shenyang, China

**Keywords:** osteoarthritis, resveratrol, chondrocytes, inflammation, apoptosis, tissue engineering, knee

## Abstract

Osteoarthritis (OA) is one of the progressing chronic joint associated with by many complex factors such as age, obesity, and trauma. Knee osteoarthritis (KOA) is the most common type of OA. KOA is characterized by articular cartilage destruction and degeneration, synovial inflammation, and abnormal subchondral bone changes. To date, no practical clinical approach has been able to modify the pathological progression of KOA. Drug therapy is limited to pain control and may lead to serious side effects when taken for a long time. Therefore, searching for safer and more reliable treatments has become necessary. Interestingly, more and more research has focused on natural products, and monomeric compounds derived from natural products have received much attention as drug candidates for KOA treatment. Resveratrol (RES), a natural phenolic compound, has various pharmacological and biological activities, including anti-cancer, anti-apoptotic, and anti-decay. Recently, studies on the effects of RES on maintaining the normal homeostasis of chondrocytes in KOA have received increasing attention, which seems to be attributed to the multi-targeted effects of RES on chondrocyte function. This review summarizes preclinical trials, clinical trials, and emerging tissue engineering studies of RES for KOA and discusses the specific mechanisms by which RES alleviates KOA. A better understanding of the pharmacological role of RES in KOA could provide clinical implications for intervention in the development of KOA.

## Introduction

Knee osteoarthritis (KOA) is a common degenerative joint disease with by many complex factors such as age, obesity, and trauma (Cushnaghan and Dieppe, 1991; Lawrence et al., 2008; Ashford and Williard, 2014). KOA is usually featured by the destruction of articular cartilage, synovial inflammation, and abnormal growth of joint margins and subchondral bone (Felson et al., 2000). KOA often causes pain and physical disability and affects millions of people aged 60 and older (Hochberg et al., 2013). More than 7% (528 million) of the world’s population is affected, and the prevalence may be higher in countries or regions with more aging populations and higher rates of obesity (Leifer et al., 2022). Presently, the primary treatment modalities for KOA include drug therapy (systemic administration and intra-articular administration) and prophylactic measures (such as exercise) to improve patients’ quality of life (Dieppe and Lohmander, 2005; Gay et al., 2016). Among them, non-steroidal anti-inflammatory drugs (NSAIDs) and acetaminophen are the more common drugs, mainly used to relieve pain and inflammation in KOA patients. However, long-term use of NSAIDs may have side effects, including gastrointestinal ulcers and bleeding, liver and cardiac and renal adverse effects (Taruc-Uy and Lynch, 2013; Rodriguez-Merchan, 2018). After these treatment options fail, arthroplasty is a standard treatment for patients with advanced KOA, but the cost is high and postoperative rehabilitation and limited prosthetic life are uncertain issues (Onggo et al., 2019). Therefore, finding safer and more effective treatments is essential to alleviate cartilage degeneration.

Natural plant compounds are widely distributed and economical and are popular among researchers for their broad range of pharmacological activities. The monomer is an active substance with a specific spatial structure separated from natural plants and is being increasingly used by researchers to study diseases (Zhou H. et al., 2021). Resveratrol (3,4′,5-trihydroxy-stilbene, RES) is a natural phenolic compound generally existing in common foods such as berries, grapes, and mulberries (Burns et al., 2002; Stervbo et al., 2007; Sales and Resurreccion, 2014). Studies show that RES is considered to have preventive and curative effects on neurodegenerative diseases, diabetes, obesity, atherosclerosis, and other diseases due to its extensive biological activities (Anekonda, 2006; Sharma et al., 2007a; Sharma et al., 2007b; Vingtdeux et al., 2008; Springer and Moco, 2019). Due to its effectiveness in disease prevention and treatment, RES has been widely used as an alternative drug in KOA treatment and has received growing interest from researchers (Kiselev, 2011). However, relatively few studies on the molecular mechanisms of RES on KOA chondrocytes. This paper summarizes the research results of the therapeutic effect of RES on KOA and discusses the specific mechanism of RES in relieving KOA better to understand the pharmacological effect of RES in KOA and provide clinical significance for intervention in the development of KOA.

## Basic Overview of RES (Structure, Bioavailability, and Biosynthesis)

**FIGURE 1:**
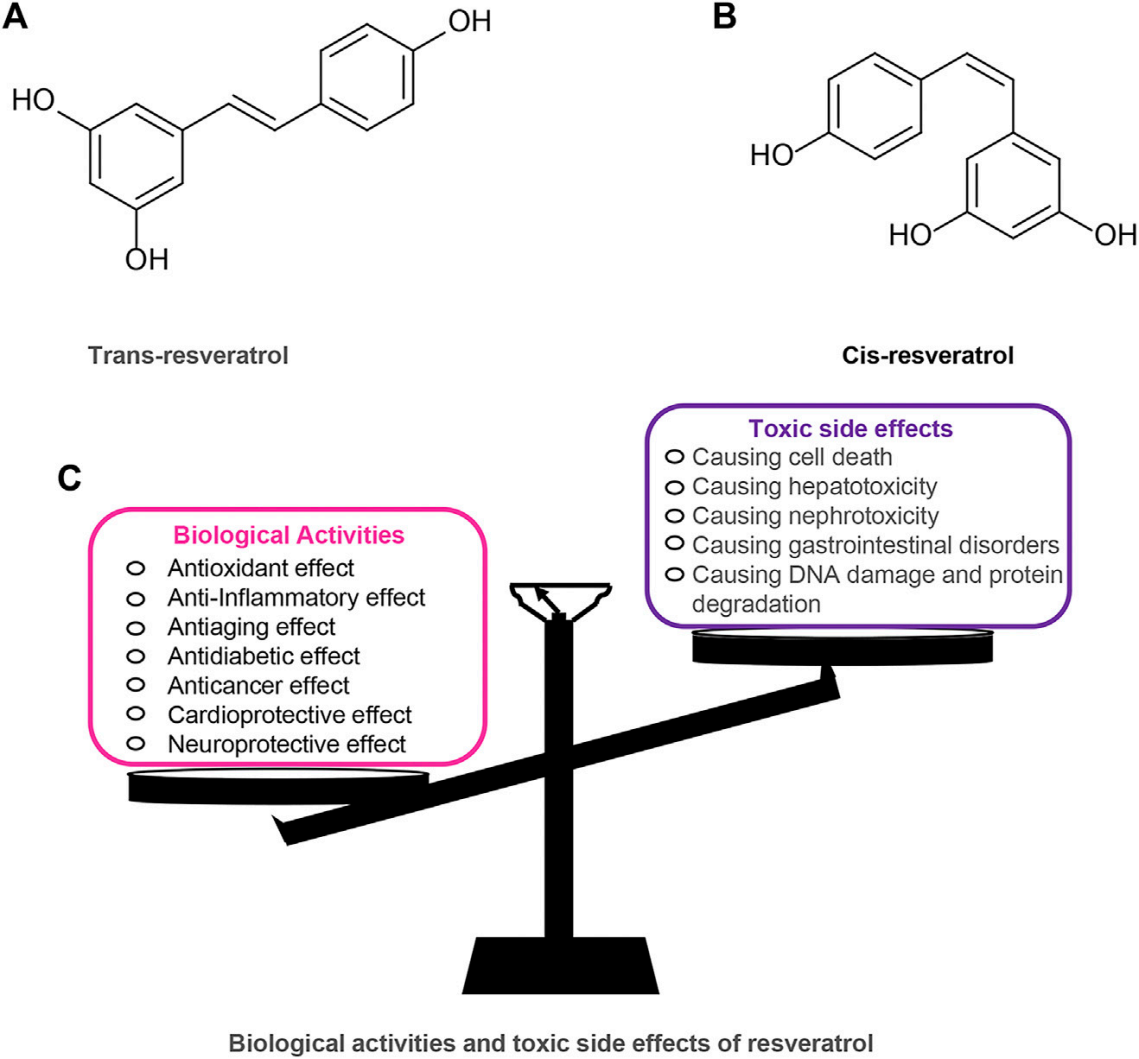
Resveratrol chemical structures (trans and cis forms) and its biological activities and toxic side effects. **(A)** Trans-resveratrol. **(B)** Cis-resveratrol. **(C)** Biological activities and toxic side effects of resveratrol.

RES has a long history and is one of the most widely studied plant metabolites. RES was first extracted from *Veratrum Grandiflorum* by Takaoka in 1939 and was widely found in more than 70 plants (Takaoka, 1939). RES has attracted much attention because of its extensive pharmacological and biological activities, such as antioxidant, antitumor, anti-aging, cardiovascular protection, and hypoglycemia (Bo et al., 2013; Andrade et al., 2018; Colica et al., 2018; Rauf et al., 2018; Pannu and Bhatnagar, 2019). Among them, RES, as a plant antioxidant, has chemopreventive and therapeutic effects on diseases such as cancer and diabetes (Abdali et al., 2015; Rauf et al., 2018). Furthermore, RES is included as part of the “French paradox” because of its ability to reduce the incidence of cardiovascular disease (Renaud and de Lorgeril, 1992). These beneficial effects of RES on human health have been reported to be closely related to its molecular structure. RES exists in nature in two isomeric forms: *trans*-Res(t-RES) and *cis*-Res(c-RES), of which the t-RES is the predominant form and is considered to have the most potent therapeutic benefits (Cardile et al., 2007; Delmas et al., 2011; Weiskirchen and Weiskirchen, 2016; Pujara et al., 2017). The cis-isomerization can occur when the trans-isomer is exposed to UV or artificial light (Camont et al., 2009). RES appears to be safe as a dietary plant-based polyphenol, but studies have also covered the toxic effects of RES utilization. Research shows that an intake range of 700–1,000 mg/kg/day of RES in rats is safe and non-toxic (Vang et al., 2011). However, in a study of healthy subjects, oral RES (2000 ml twice daily) was found to trigger mild diarrhea in the subjects (la Porte et al., 2010). Furthermore, some *in vitro* studies have shown that RES can exhibit a biphasic dose-effect. In ethanol-induced cell death models, RES is protective at low doses (10–25 µM) and prevents ethanol-induced apoptosis but toxic at high doses (100–200 µM) (Chan and Chang, 2006). (Figure 1).

Additionally, the efficacy of RES is limited by its low oral bioavailability, resulting from low absorption rates in the gastrointestinal tract, rapid metabolism, and rapid excretion (Soleas et al., 2001; Walle et al., 2004; Chen et al., 2013). After RES is ingested into the gastrointestinal tract, it is absorbed by diffusion across the epithelium or by binding to transporter proteins on cell membranes to form complexes with a high absorption rate. These complexes follow the blood flow to all parts of the body, and when they encounter cells with lipoprotein and albumin receptors, RES dissociates into its free form and exerts its physiological effects. Consequently, these compounds function as reservoirs for RES and assist in its travel (Delmas et al., 2011). Besides, RES has a high metabolic capacity. RES generates glucuronide and sulfate in intestinal epithelial cells by interacting with UDP-glucuronosyltransferase or sulfate transferase (Chen et al., 2013). It is worth mentioning that RES displays a good effect *in vivo*, probably due to the reconversion of glucuronide and sulfate to RES in target organs such as the liver to improve its bioavailability (Vitrac et al., 2003; Wenzel and Somoza, 2005). At the same time, RES goes through a fast excretion process in the body. After being rapidly absorbed by the gastrointestinal tract, most RES is excreted through the urinary tract (Nunes et al., 2018). To address these issues, researchers have found that the ground bioavailability of RES can be addressed by micelles, liposomes, and nanoparticles (Tsai et al., 2012). For instance, Singh et al. discovered that encapsulation of RES in polymeric nanocarriers of poly (lactic acid)-hydroxyacetic acid copolymer (PLGA) significantly improved the bioavailability of RES (Singh and Pai, 2014). In addition, various studies have shown that RES can potentially enhance the chemopreventive effects through binding to other phytochemicals (e.g., piperine) or synthesizing derivatives of RES to increase bioavailability (Asensi et al., 2002). For example, a methylated derivative of RES (3,4,5,4’trimethoxystilbene) effectively prevents colon cancer in a rat model with improved bioavailability (Lin et al., 2009).

Due to the health effects of RES on humans, its production in large quantities is necessary. Currently, commercial bulk extraction of RES is challenging to achieve because of its low concentration, complex separation and purification steps, and seasonal limitations (Mei et al., 2015). Therefore, a large amount of research has turned to find alternative methods. Currently, many biotechnological methods, such as genetic engineering and tissue culture, have been used as alternative methods for RES production (Nandagopal et al., 2018). For example, Kiselev et al. developed the extraction of RES from transgenic cell cultures of V. *amurensis* transformed with a single *rol*B gene (Kiselev et al., 2007).

## Preclinical Studies About RES and its Effects in KOA

**FIGURE 2:**
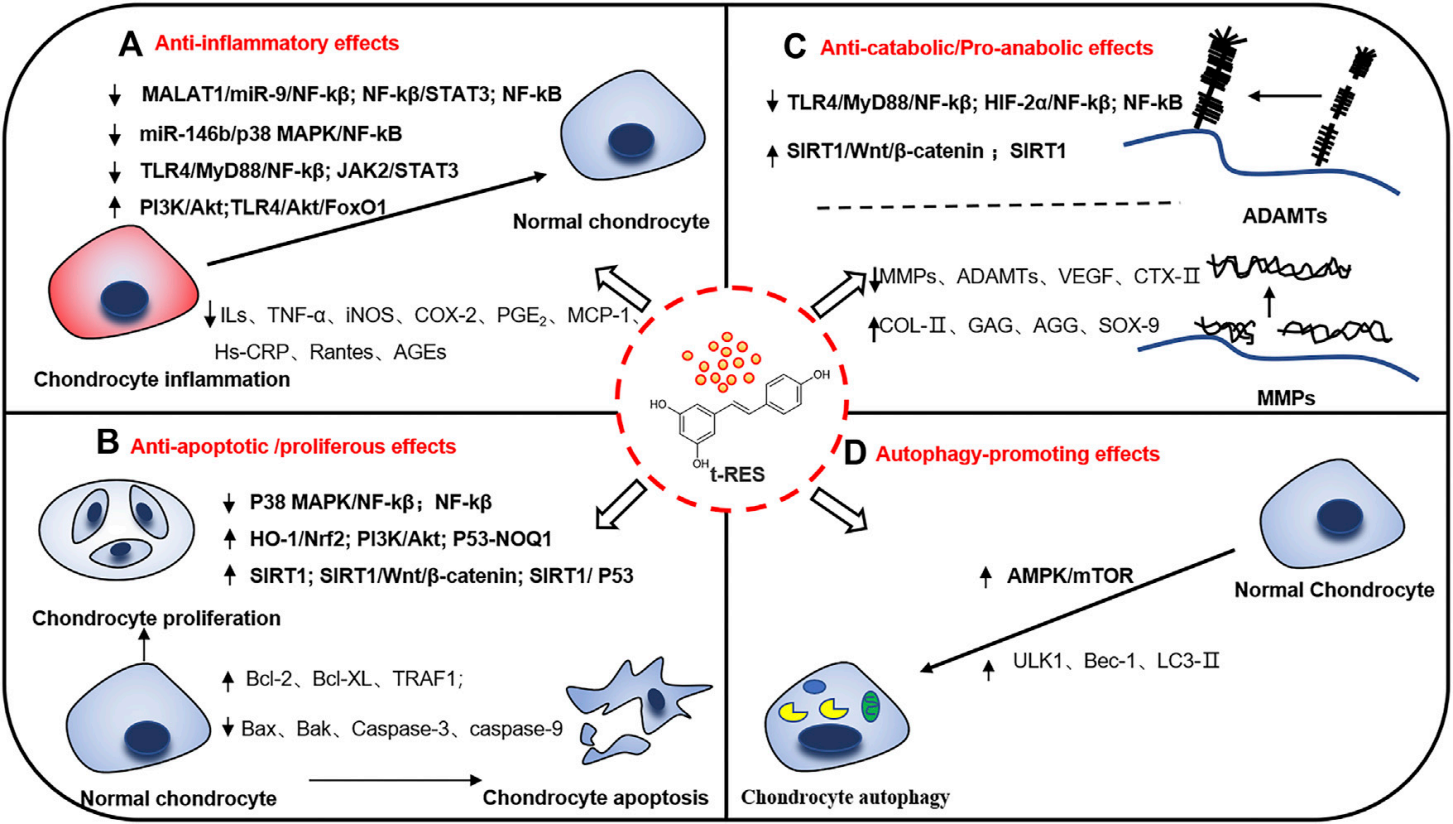
Potential mechanisms of resveratrol in relieving knee osteoarthritis. There are many molecular mechanisms involved in the occurrence and development of knee osteoarthritis. Resveratrol plays multiple positive roles in knee osteoarthritis, reducing inflammatory activation, cell apoptosis, maintaining cartilage homeostasis and promoting autophagy through several signal pathways. **(A)** Anti-inflammatory effects. **(B)** Anti-apoptotic/proliferous effects. **(C)** Anti-catabolic/Pro-anabolic effects. **(D)** Autophagy-promoting effects. ↑: up-regulation. ↓: down-regulation.

Recently, numerous studies have indicated that RES may postpone the progression of KOA through anti-inflammation, anti-apoptosis, maintenance of cartilage homeostasis, and promotion of cellular autophagy. Therefore, RES may be a promising complementary and alternative therapy for KOA with extensive interest from researchers. This review discusses recent research on RES-mediated related molecular mechanisms and signaling pathways to treat KOA. The possible role of RES in mitigating KOA and its corresponding mechanisms will be described in the following subsection. (Figure 2). Besides the following details, the results of preclinical studies of RES for KOA are shown in Table 1.

**TABLE 1:** Preclinical studies about RES and its effects on KOA.

Main effects	Mechanisms	Study type	Dosage range	Model (Animals/Cells)	References
Anti-inflammatory effects					
	-Decreased IL-1β and TNF-α levels	*In vitro* study	Resveratrol and vitamin E incubation (5–200 µM) for 2 h	H_2_O_2_-induced porcine chondrocytes	Su et al. (2021)
	-Decreased MMP-1 and MMP-13 levels				
	-Suppressed COX-2/iNOS signaling pathway	*In vivo* study	Resveratrol oral administration (5 or 10 mg/kg/day) for 2 weeks	MIA-induced KOA SD rats	Wang et al. (2016)
	-Decreased IL-1β, IL-6, TNF-α, and MMP-13 levels				
	-Increased IL-6 levels	*In vitro* study	Resveratrol incubation (0–5 µM) for 48 h	PBMC in KOA patients	Wendling et al. (2013)
	- Decreased TNF-α, IL-6, and hs-CRP levels	*In vivo* study	Resveratrol oral administration (30 mg/kg/day) for 12 weeks	T2DM-induced KOA rats	Ebrahim et al. (2020)
	- Decreased glucose and glycosylated hemoglobin				
	-Improved lipid profile (TG, CHOL, LDL-C, and HDL-C)				
	- Decreased glycemia and dyslipidemia	*In vivo* study	Resveratrol oral administration (30 mg/kg/day) for 12 weeks	T2DM-induced KOA rats	El-Bidawy et al. (2021)
	-Decreased the blood levels of HbA1c, TG, CHOL, and LDL-C				
	-Inhibited IL-6, TNF-α, and hs-CRP levels				
	-Inhibited the combination of AGEs and RAGE	*In vitro* study	Resveratrol and curcumin incubation (12–500 μM) for 8 days	IL-1β and AGE modified bovine serum albumin-induced cartilage	Mehta et al. (2021)
	-Suppressed NF-kB signaling pathway	*In vitro* study	Resveratrol and curcumin incubation (50 μM) for 4 or 24 h	IL-1β-induced human chondrocytes	Csaki et al. (2009)
	-Lower COX-2, MMP-3, MMP-9, and VEGF levels				
	-Suppressed NF-kB signaling pathway	*In vitro* study	Resveratrol incubation (6, 12, 24, and 48 μM) for 24 h	IL-1β-induced human chondrocytes	Yi et al. (2020)
	-Inhibited IL-1β-induced iNOS, NO, COX-2, and PGE_2_ levels				
	-Suppressed NF-kB/STAT3 signaling pathway	*In vitro* study	Resveratrol incubation (10, 25, 50, and 100 μM) for 24 h	IL-1β-induced human chondrocytes and macrophages	Limagne et al. (2016)
	-Decreased IL-6, IL-8, MCP-1, and RANTES levels				
	-Activated HO-1/Nrf2 signaling pathways	*In vivo* study	Resveratrol (50 mg/kg/3 days) for 8 weeks	MIA-induced KOA Wistar rat	Wei et al. (2018)
	-Suppressed NF-kB signaling pathway				
	-Decreased TNF-α, IL-1β, IL-6, and IL-18 levels				
	-Suppressed iNOS and SIRT1 expressions				
	-Suppressed TLR4/MyD88/NF-kB signaling pathway	*In vitro* study	Resveratrol incubation (0–200 µM) for 24 h	IL-1β-induced human chondrocytes	Liu et al. (2014)
	-Decreased IL-1β level				
	-Suppressed TLR4/MyD88 signaling pathway	*In vitro* study	Resveratrol incubation (0, 6.25, 12.5, 25, 50, 100, and 200 µM) for 24 h	IL-1β-induced human chondrocytes	Gu et al. (2017)
	-Inhibited IL-6 and MMP-13 levels				
	- Suppressed TLR4 signaling pathway	*In vitro* and *vivo* study	Resveratrol incubation (50 µM) for 30 min or 24 h Resveratrol oral administration (45 mg/kg/day) for 8 weeks	IL-1β-induced SW1353 cells/HFD-induced KOA C57BL/6J mice	Xu et al. (2019)
	-Activated PI3K/Akt signaling pathway				
	-Increased p-PI3K and p-AKT levels				
	-Suppressed TLR4/Syk/NF-kB signaling pathway	*In vitro* study	Resveratrol incubation (2–8 μmol/L) for 48 h	IL-1β-induced rat chondrocyte	Ma et al. (2020)
	-Inhibited iNOS, NO, COX-2, and PGE_2_ and COX-2 levels				
	-Increased IL-6, IL-17, and TNF-α levels				
	-Enhanced TLR4/Akt/FoxO1 self-limiting axis	*In vitro* study	Resveratrol incubation (500 μM) for 30 min or 24 h	IL-1β-induced SW1353 cells	Xu et al. (2020)
	-Suppressed TLR4/MyD88/NF-kB signaling pathway				
	-Inhibited IL-6 levels				
	-Suppressed TLR4/TRAF6 signaling pathways	*In vivo* study	Resveratrol oral administration (22.5 or 45 mg/kg/day) for 12 weeks	HFD-induced KOA C57BL/6J mice	Jiang et al. (2017)
	-Decreased IL-1β and leptin levels				
	-Suppressed TLR4/MyD88/NF-kB signaling pathways	*In vivo* study	Resveratrol oral administration (40 or 80 mg/kg/day) for 4 weeks	Surgery-induced SD rats	Long et al. (2021)
	-Decreased IL-1β, IL-6, TNF-α, and MCP-1 levels				
	-Suppressed JAK2/STAT3 signaling pathway	*In vitro* and *vivo* study	Resveratrol incubation (50 μM) for 2 or 24 h Resveratrol oral administration (45 mg/kg/day) for 22 weeks	Leptin-induced mice chondrocytes and SW1353 cells HFD-induced KOA C57BL/6J mice	Jiang et al. (2020)
	-Reduced MMP-13 and leptin levels				
	-Down-regulated SOCS3 levels				
	-Activated PI3K/Akt signaling pathway	*In vitro* study	Resveratrol incubation (3.125–100 μmol/L) for 24 or 48 h	IL-1β-induced rat chondrocytes	Liu and Lv, (2019)
	-Decreased TNF-α and IL-6 levels				
	-Inactivated p38 MAPK and NF-kB signaling pathway	*In vitro* study	Resveratrol incubation (0–50 µM) for 24 h	LPS-induced ATDC5 cells	Jin et al. (2018)
	-Decrease IL-1β, IL-6, and TNF-α levels				
	-Activated p38 MAPK and ERK1/2 phosphorylation	*In vitro* study	Resveratrol incubation (100 nM) for 24 h	IS526-induced rabbit articular chondrocytes	Eo and Kim, (2020b)
	-Increased COX-2 and PGE_2_ levels				
Anti-apoptotic effects					
	-Suppressed NF-kB signaling pathway	*In vitro* study	Resveratrol and curcumin incubation (50 μM) for 4 or 24 h	IL-1β-induced human chondrocytes	Csaki et al. (2009)
	-Increased Bcl-2, Bcl-xl, and TRAF1 levels				
	-Inhibited caspase-3 levels and PARP cleavage				
	-Activated p53-NQO1 signaling pathway	*In vitro* study	Resveratrol incubation (100 μM) for 1, 12, 24, 36, and 48 h	IL-1β-induced primary human chondrocytes	Csaki et al. (2008)
	-Inhibited caspase-3 and PARP cleavage				
	-Suppressed ROS levels				
	-Suppressed MALAT1/miR-9/NF-kB signaling pathways	*In vitro* and *vivo* study	Resveratrol incubation (15 or 30 μM) for 24, 48, 72, and 96 h Resveratrol injection (10 ml/2 days) for 8 weeks	KOA mice chondrocytes DMM-induced KOA C57BL/6J mice	Zhang et al. (2020)
	-Decreased IL-6, MMP-13, and caspase-3 levels				
	-Suppressed TLR4/NF-kB signaling pathway	*In vivo* study	Resveratrol oral administration (40 or 80 mg/kg/day) for 4 weeks	Surgery-induced SD rats	Long et al. (2021)
	-Decreased Bax and caspase-9 levels				
	-Increased Bcl-2 level				
	-Decreased caspase-3 levels	*In vitro* study	Resveratrol incubation (100 μM) for 1, 2, and 24 h	IL-1β-induced human chondrocytes	Shakibaei et al. (2007)
	-Inhibited PARP cleavage				
	-Inhibited COX-2 and PGE_2_ levels	*In vitro* study	Resveratrol incubation (1–10 μM) for 1 h	IL-1β-induced human chondrocytes	Dave et al. (2008)
	-Reduced cytochrome c levels				
	-Regulated actin organization	*In vitro* study	Resveratrol incubation (100 μM) for 24 h	SNP-induced rabbit chondrocytes	Jin et al. (2014)
	-Decreased ROS levels	*In vitro* study	Resveratrol incubation (100 μM) for 12, 18, and 24 h	SNP-induced rabbit chondrocytes	Liang et al. (2014)
	-Inhibited Bax and Bak levels				
	-Decrease NO levels	*In vivo* study	Resveratrol injection (10 or 20 or 50 μmol/kg/day) for 2 weeks	Hulth-Telhag-induced KOA New Zealand white rabbits	Wang et al. (2012)
	-Lowered TUNEL-positive cells levels	*In vivo* study	Resveratrol oral administration (5, 22.5 or 45 mg/kg/day) for 12 weeks	HFD-induced KOA C57BL/6J mice	Gu et al. (2016)
	-Upregulated LINC00654 levels	*In vitro* study	Resveratrol incubation (25 μM) for 2 h	IL-1β-induced human chondrocytes	Yi et al. (2021)
	-Downregulated miR-210-5p levels				
	-Decreased chondrocytes apoptotic ratio				
	-Suppressed SIRT1/Wnt/β-catenin signaling pathway	*In vitro* study	Resveratrol incubation (10 µM) for 48 h	IL-1β-induced human chondrocytes	Liu et al. (2017)
	-Decreased Bax, caspase-3, and caspase-9 levels				
	-Increased Bcl-2 levels				
	-Activated SIRT1/NF-kB signaling pathway	*In vitro* study	Resveratrol incubation (0–20 μM) for 1 or 9 h	Il-1β-induced human chondrocytes	Lei et al. (2012)
	-Decreased iNOS and NO expressions				
	-Suppressed SIRT1/P53 signaling pathway	*In vivo* study	Resveratrol injection (5, 10 or 15 μmol/L) for 6 weeks	DMM-induced New Zealand white rabbits	Zhenquan Zhou et al. (2021)
	-Decreased iNOS and NO expressions				
	-Activated PI3K/Akt signaling pathway	*In vitro* study	Resveratrol incubation (3.125–100 μmol/L) for 24 or 48 h	IL-1β-induced rat chondrocytes	Liu and Lv, (2019)
	-Decreased Bax and caspase-3 levels				
	-Increased Bcl-2 level				
	-Inactivated p38 MAPK and NF-kB signaling pathway	*In vitro* study	Resveratrol incubation (0–50 µM) for 24 h	LPS-induced ATDC5 cells	Jin et al. (2018)
	-Decreased Bax and caspase-3 levels				
	-Increased Bcl-2 levels				
	-Activated MAPK/ERK signaling pathway	*In vitro* study	Resveratrol and curcumin incubation (10 μM) for 4 or 24 h	Il-1β-induced human chondrocytes	Shakibaei et al. (2011)
	-Decreased caspase-3 levels				
	-Activated HO-1/Nrf2 signaling pathways	*In vivo* study	Resveratrol (50 mg/kg/3 days) for 8 weeks	MIA-induced KOA Wistar rat	Wei et al. (2018)
	-Decreased caspase-3 and caspase-9 levels				
Maintaining cartilage homeostasis effects					
	-Suppressed NF-kB signaling pathway	*In vitro* study	Resveratrol and curcumin incubation (50 μM) for 4 or 24 h	IL-1β-induced human chondrocytes	Csaki et al. (2009)
	-Lower COX-2, MMP-3, MMP-9, and VEGF levels				
	-Increased COL-Ⅱ and SOX-9 levels				
	-Inhibited GAG depletion and MMP-13 levels	*In vitro* study	Resveratrol incubation (50 μM) for 2 weeks Resveratrol preincubation (50 μM) for 1 week	Il-1β-induced porcine chondrocytes	Frischholz et al. (2020)
	-Decreased COL-Ⅰ levels				
	-Increased COL-Ⅱ and COL-X levels				
	-Suppressed TLR4/Syk/NF-kB signaling pathway	*In vitro* study	Resveratrol incubation (2–8 μmol/L) for 48 h	IL-1β-induced rat chondrocyte	Ma et al. (2020)
	-Decreased MMP-9 and MMP-13 levels				
	-Increased GAG and COL-Ⅱ expressions				
	-Suppressed NF-kB signaling pathway	*In vitro* study	Resveratrol incubation (6, 12, 24, and 48 μM) for 24 h	IL-1β-induced human chondrocytes	Yi et al. (2020)
	-Increased COL-Ⅱ level				
	-Increased COL-Ⅱ levels	*In vitro* study	Resveratrol incubation (20, 50, and 100 μM) for 24 h	Porcine chondrocytes	Maepa et al. (2016)
	-Decreased MMP-1 and MMP-13 levels	*In vitro* study	Resveratrol and vitamin E incubation (5–200 µM) for 2 h	H_2_O_2_-induced porcine chondrocytes	Su et al. (2021)
	-Increased COL-Ⅱ expression				
	-Decreased MMP-1, MMP-3, and MMP-13 levels	*In vitro* study	Resveratrol incubation (1–10 μM) for 1 h	IL-1β-induced human chondrocytes	Dave et al. (2008)
	-Suppressed NF-kB signaling pathway	*In vivo* study	Resveratrol injection (10μMol/kg/day) for 2 weeks	ACLT-induced KOA Twelve New Zealand white rabbit model	Elmali et al. (2005)
	-Inhibited matrix proteoglycan loss				
	-Decreased CTX-Ⅱ level	*In vivo* study	Resveratrol oral administration (5, 22.5 or 45 mg/kg/day) for 12 weeks	HFD-induced KOA C57BL/6J mice	Gu et al. (2016)
	-Suppressed MAPK/AP-1 and IKK/NF-kB signaling pathways	*In vitro* study	Resveratrol incubation (25, 50, 75, and 100 μM) for 48 h	AGEs-induced porcine chondrocytes	Liu et al. (2010)
	-Decreased MMP-13 level				
	-Increased COL-Ⅱ and AGG levels				
	-Inhibited GAG loss	*In vitro* study	Resveratrol and curcumin incubation (12–500 μM) for 8 days	IL-1β and AGE modified bovine serum albumin-induced cartilage	Mehta et al. (2021)
	-Suppressed NF-kB signaling pathway	*In vitro* study	Resveratrol incubation (1–100 μM) for 24 h	IL-1β-induced rabbit chondrocytes and SW1353 cells	Kang et al. (2018)
	-Decreased MMP-1, MMP-3, MMP-13, ADAMTS-4, and ADAMTS-5 levels				
	-Suppressed TLR4/MyD88 signaling pathway	*In vitro* study	Resveratrol incubation (0, 6.25, 12.5, 25, 50, 100, and 200 µM) for 24 h	IL-1β-induced human chondrocytes	Gu et al. (2017)
	-Inhibited IL-6 and MMP-13 levels				
	-Suppressed SIRT1/Wnt/β-catenin signaling pathway	*In vitro* study	Resveratrol incubation (10 µM) for 48 h	IL-1β-induced human chondrocytes	Liu et al. (2017)
	-Decreased MMP-1, MMP-3, MMP-13, Wnt3a, Wnt5a, Wnt7a, and β-catenin levels				
	-Suppressed NF-kB/HIF-2α signaling pathway	*In vivo* study	Resveratrol injection (10 or 100 μg) for 4 weeks	DMM-induced KOA C57BL/6 mice	Li et al. (2015)
	-Increased SIRT1 expression				
	-Increased COL-Ⅱ expression				
	-Increased SIRT1 expression	*In vitro* study	Resveratrol incubation (1–50 μM) for 24, 48, 72 or 6 weeks	KOA human chondrocytes	Kim et al. (2014)
	-Increased COL-Ⅹ, AGG, and RUNX2 levels				
	-Activated ERK1/2 and PIK3/Akt signaling pathway	*In vitro* study	IS526 (12.3 ng/ml) for 24 h	Primary rabbit chondrocytes	Eo and Kim, (2020a)
	-Decreased COL-Ⅱ and SOX-9 levels				
	-Increased MMP-13 expression				
Promoting autophagy effects					
	-Activated AMPK/mTOR signaling pathway	*In vivo* study	Resveratrol injection (10 ml/kg/2 days) for 8 weeks	DMM-induced KOA C57BL/6 mice	Qin et al. (2017)
	-Increased ULK1, Bel1, and LC3-Ⅱ levels				
	-Decreased HIF-2α levels				
	-Increased HIF-1α levels				

Abbreviations: **RES**, resveratrol; **KOA**, knee osteoarthritis; **IL-1β**, interleukins-1β; **TNF-α**, tumor necrosis factor-α; **MMP-1**, matrix metalloproteinase-1; **MMP-13**, matrix metalloproteinase-13; **H**_**2**_ **O**_**2**_, hydrogen peroxide; **IL-6**, interleukins-6; **MIA**, sodium iodoacetate; **SD**, Sprague Dawley; **PBMC**, peripheral blood mononuclear cells; **hs-CRP**, high-sensitivity C-reactive protein; **TG**, triglyceride; **CHOL**, total cholesterol; **LDL-C**, low density lipoprotein-Cholesterol; **HDL-C**, high density lipoprotein-cholesterol; **HbA1c**, high blood levels of glycated hemoglobin; **T2DM**, diabetes mellitus type 2; **AGEs**, advanced glycosylation end products; **RAGE**, receptor for AGE; **NF-kB**, nuclear factor-kappa B; **COX-2**, cyclooxygenase-2; **MMP-3**, matrix metalloproteinase-3; **MMP-9**, matrix metalloproteinase-1; **VEGF**, vascular endothelial growth factor; **PGE**_**2**_, prostaglandin E2; **STAT3**, signal transduction and activator of transcription 3; **MCP-1**, monocyte chemoattractant protein-1; **Rantes**, regulated upon activation normal T cell expressed and secreted factor; **IL-8**, interleukins-8; **HO-1**, heme oxygenase 1; **Nrf2**, nuclear factor erythroid 2-related factor 2; **IL-18**, interleukin 18; **iNOS**, nitric oxide synthase; **SIRT1**, Sirtuin1; **TLR4**, toll-like receptor 4; **MyD88**, myeloid differentiation factor 88; **PI3K**, phosphatidylinositol-3-kinase; **Akt**, protein kinase B; **HFD**, high-fat diet; **Syk**, spleen tyrosine kinase; **NO**, nitric oxide; **IL-17**, interleukins-17; **FoxO1**, Forkhead box O1; **TRAF6**, TNF receptor-associated factor 6; **JAK2**, janus kinase 2; **ROS**, reactive oxygen species;**SOCS3**, suppressor of cytokine signaling 3; **p38 MAPK**, p38 mitogen activated protein kinase; **LPS,** lipopolysaccharide**; ERK1/2**, extracellular regulated protein kinases 1/2;**Bcl-2**, B-cell lymphoma-2; **Bcl-XL**, B-cell lymphoma-XL; **TRAF1**, tumor necrosis factor-α receptor-associated factor 1; **caspase-3**, cysteine aspartate protease-3; **PARP**, poly (ADP-Ribose) polymerase; **NQO1,** NAD(P)H: quinon oxidor-eductase; **MALAT1**, metastasis-associated lung adenocarcinoma transcript 1; **DMM**, destabilizing medial meniscus; **Bax**, Bcl-associated X; **Bak**, Bcl-associated K; **SNP**, sodium nitroprusside; **MAPK**, mitogen activated protein kinase; **COL-Ⅱ**, type II collagen; **SOX-9**, cartilage-specific transcription factor 9; **GAG**, glycosaminoglycan; **COL-Ⅰ**, type Ⅰ collagen; **COL-Ⅹ**, type Ⅹ collagen; **CTX-Ⅱ**, C-telopeptide of type Ⅱ collagen; **ACLT**, anterior cruciate ligament transection; **AP-1**, activator protein-1; **AGG**, aggregate; **ADAMTs-4**, a disintegrin and metalloproteinase with thrombospondin motifs-4; **ADAMTs-5**, a disintegrin and metalloproteinase with thrombospondin motifs-5; **RUNX2**, runt-related transcription factor 2**AMPK**, AMP activated protein kinase; **mTOR**, mammalian target of rapamycin; **HIF-2α**, hypoxia inducible factor-2α; **HIF-1α**, hypoxia inducible factor-1α; **ULK1**, Unc-51elike kinase1; **LC3-Ⅱ**, microtubule-associated protein light chain 3; **Bec-1**, beclin 1.

### RES and its Anti-Inflammatory Effects in KOA

KOA is influenced by several pathogenic factors, among which inflammation is considered the key factors involved in the development and progression of KOA. Elevated levels of inflammatory agents are closely associated with KOA progression (Shen et al., 2017; Ansari et al., 2020). Studies show that under pathological conditions of KOA, chondrocytes and synoviocytes overexpress inflammatory factors, such as interleukin-1β (IL-1β), cyclooxygenase 2(COX-2), and tumor necrosis factor-α (TNF-α), to hasten cartilage degeneration (Kapoor et al., 2011). RES is widely used to treat inflammatory diseases because of its good anti-inflammatory activity (Wang et al., 2018; Yuan et al., 2019; Zheng and Chen, 2021). Several studies show that RES can improve KOA inflammation by modulating inflammatory factors in some traditional KOA models. Su et al. found that RES pretreatment of KOA chondrocytes significantly ameliorated the hydrogen peroxide (H_2_O_2_)-induced inflammatory response (Su et al., 2021). In further animal studies, Wang et al. found that RES sustained treatment for 2 weeks significantly inhibited the expression of inflammatory mediators in the sodium iodoacetate (MIA)-induced KOA rats (Wang et al., 2016). However, inconsistent with the studies mentioned above, Wendling et al. treated with RES *in vitro*, and the level of IL-6 in serum mononuclear cells of KOA patients increased in a dose-dependent manner (Wendling et al., 2013). This suggests that RES may have different effects in different *in vitro* chondrocyte models. The reason for this may be due to the variability in the concentration of RES administered in different chondrocyte types. Besides, recent studies have shown that advanced glycosylation end products (AGEs) are also engaged in KOA inflammation, and that AGEs binding to the receptor for AGEs (RAGE) has been shown to upregulate inflammatory markers to exacerbate cartilage inflammatory responses (Yang et al., 2011; Vistoli et al., 2013). Several studies have found that RES treatment for 12 weeks in a diabetes mellitus type 2 (T2DM)-induced KOA rat model significantly improved the inflammatory response of cartilage in the high-glucose state as well as abnormal blood glucose levels (Ebrahim et al., 2020; El-Bidawy et al., 2021). Besides, RES and curcumin supplementation prevented AGEs-induced cartilage inflammatory response by inhibiting AGEs accumulation, probably due to the unique chemical structure of RES and curcumin, both of which have p-hydroxyl groups in their structure that competitively inhibit the binding of AGEs to RAGE (Mehta et al., 2021). However, a further demonstration of the inhibitory effect of RES alone on AGEs-induced inflammation is still needed. However, at this research stage, it is still impossible to determine how RES improves the mechanism of action of KOA explicitly by modulating AGEs. Further studies are still needed to confirm the inhibitory effect of RES alone on AGEs-induced inflammation.

Moreover, RES also treats KOA by mediating inflammation-related signaling pathways, which has been well demonstrated *in vitro* and *in vivo* studies. Among them, nuclear factor-kB (NF-kB) is involved in KOA development mainly through mediating inflammation-related pathways (Jimi et al., 2019). Several studies have shown that the anti-inflammatory effect of RES is mainly through modulation of the NF-kB pathway to treat KOA. For example, RES was found to reduce the inflammatory response in chondrocytes, with a possible mechanism being through partial inhibition of the IL-1β-induced NF-kB pathway (Csaki et al., 2009; Yi et al., 2020). To better mimic the environment in which KOA occurs, Limagne et al. co-exposed chondrocytes and macrophages to an inflammatory environment and observed that RES disrupted the inflammatory expansion of NF-kB/STAT3 between chondrocytes and macrophages (Limagne et al., 2016). In addition, Wei et al. demonstrated in a KOA rat model that RES played a role in alleviating inflammatory damage and preventing KOA by inhibiting the NF-kB pathway (Wei et al., 2018). Besides, the toll-like receptor 4 (TLR4) pathway performs a crucial function in obesity-associated KOA, a condition believed to be engaged in systemic low-density inflammation. RES may act as an anti- KOA agent by modulating the TLR4-related pathway to improve the status of KOA chondrocytes (Zeddou, 2019). Several *in vitro* studies have shown that RES downregulates TLR4 expression and ameliorates cartilage inflammatory responses through the TLR4/myeloid differentiation factor 88 (MyD88)/NF-kB pathway. Furthermore, RES dimer amurensin H suppressed inflammation by inhibiting TLR4/spleen tyrosine kinase (Syk) signaling pathways (Liu et al., 2014; Gu et al., 2017; Xu et al., 2019; Ma et al., 2020). Similarly, Xu et al. first found RES in IL-1β-stimulated SW1253 cells to attenuate the KOA inflammatory response by enhancing the TLR4/protein kinase B (Akt)/Forkhead box O1 (FoxO1) axis (Xu et al., 2020). Moreover, RES alleviated inflammation and minimized bodyweight in KOA mice via the TLR4/NF-kB pathway in a high-fat diet (HFD)-induced obesity-associated KOA mouse model (Jiang et al., 2017; Xu et al., 2019; Long et al., 2021). In another study of obesity-related KOA, Jiang found that RES attenuated obesity-related KOA by attenuating the janus kinase 2 (JAK2)/STAT3 signaling pathway independent of the suppressor of cytokine signaling 3 (SOCS3) pair (Jiang et al., 2020). Thus, the above suggests that RES may become a complementary treatment for obesity-related KOA. Furthermore, RES alleviates the inflammatory response of KOA by regulating multiple other signaling pathways that mediate inflammation. Liu et al. found that RES pretreatment significantly activated the phosphatidylinositol-3-kinase (PI3K)/Akt signaling pathway to alleviate IL-1β-induced inflammatory response in chondrocytes (Liu and Lv, 2019). Similarly, it was also found that RES could upregulate miR-146b to inactivate p38 mitogen-activated protein kinase (p38 MAPK) and NF-kB signaling pathways to protect mouse ATDC5 cells from IL-1β-induced inflammatory damage (Jin et al., 2018). However, Eo et al. used a RES-rich extract (IS526) to culture chondrocytes. They showed that IS526 induced inflammation in rabbit chondrocytes through activation of the p38 MAPK and extracellular regulated protein kinases 1/2 (ERK1/2) pathways (Eo and Kim, 2020). This contrasts with the findings of Eo et al. the latter concluded that RES extract exacerbated the inflammatory response of chondrocytes, suggesting that RES may have different effects in different *in vitro* chondrocyte models.

Taken together, *in vitro* and animal models show that RES supplementation effectively improves in improving the inflammatory response in KOA. Preclinical evidence supports the therapeutic value of RES on the inflammatory response in KOA and has shown promising efficacy in different animal models (traditional-type models, obesity-related models, etc.). Thus, these studies suggest that RES may alleviate KOA through anti-inflammatory effects.

### RES and its Anti-Apoptotic Effects in KOA

Chondrocytes are the main component of cartilage tissue and are essential for maintaining cartilage health and functional integrity. Excessive apoptosis and reduced proliferation of chondrocytes are often accompanied by cartilage degeneration (Varela-Eirin et al., 2018). Recent studies have shown that RES can improve KOA chondrocytes by promoting cell proliferation. Csaki et al. found that RES and curcumin pretreatment of human chondrocytes significantly promoted chondrocyte proliferation by ameliorating the toxic effects induced by IL-1β (Csaki et al., 2009). Other studies found that RES alone dose-dependently inhibited IL-1β-stimulated reduced cell proliferation (Csaki et al., 2008; Zhang et al., 2020). However, Yi et al. exposed chondrocytes to RES at low to medium and high concentrations, respectively, in order to determine the biosafe concentration of RES. The results showed that low to medium concentrations of RES was not toxic to chondrocytes, while high doses of RES (48 μM) had significant toxic effects on chondrocytes (Yi et al., 2020). The above studies suggest that different concentrations of RES have different bioactive effects, and therefore chondrocytes should be cultured at appropriate concentrations of RES.

Apart from regulating cell proliferation, RES may also reduce cartilage degeneration by preventing chondrocyte apoptosis. Recent studies have shown that the chondroprotective effect of RES is mediated by apoptosis-related factors (Csaki et al., 2009; Long et al., 2021). Further studies found that RES pretreatment alone exerted its anti-apoptotic effect by inhibiting IL-1β-induced cleavage of caspase-3 (Shakibaei et al., 2007). Furthermore, studies have shown that IL-1β-induced apoptosis in chondrocytes is associated with their intracellular mitochondrial dysfunction. Dave et al. demonstrated that RES inhibits prostaglandin E2 (PGE_2_)-mediated mitochondrial depolarization and adenosine triphosphate (ATP) production to chondrocyte apoptosis (Dave et al., 2008). Interestingly, in sodium nitroprusside (SNP)-induced chondrocyte apoptosis model, the researchers observed that RES protected chondrocytes from apoptosis by scavenging ROS and preventing abnormal cytoskeletal remodeling, suggesting that RES has the potential to prevent changes in chondrocyte structure (Jin et al., 2014; Liang et al., 2014). Furthermore, in animal models of KOA, RES has also been shown to prevent KOA by inhibiting NO production and reducing the number of TUNEL-positive cells, thereby preventing cartilage destruction (Wang et al., 2012; Gu et al., 2016). Additionally, it has been shown that lncRNAs function in regulating KOA apoptosis and are essential targets for the anti-apoptotic activity of RES (Liu et al., 2018; Li et al., 2020; Xie et al., 2020). Yi et al. found that RES can improve apoptosis of KOA chondrocytes by regulating LINC00654, which is believed to be a prospective target for ameliorating KOA (Yi et al., 2021). The above *in vitro* evidence confirms that RES is considered a promising complementary therapeutic agent for treating KOA by acting on different *in vitro* chondrocyte models.

Furthermore, RES reduces chondrocyte apoptosis by regulating multiple signaling pathways that mediate apoptosis, thereby improving KOA. Among them, Sirtuin1 (SIRT1), as a multi-active enzyme, plays a crucial function in regulating cell proliferation and apoptosis. Therefore, RES may reduce apoptosis of KOA chondrocytes by regulating multiple signaling pathways mediated by SIRT1(Michan and Sinclair, 2007; Yamamoto et al., 2007; Seo et al., 2012). Liu et al. found that RES exerts its anti-apoptotic effects by enhancing the SIRT1/Wnt signaling pathway, inhibiting apoptosis-related molecules, and enhancing the expression of anti-apoptotic genes (Liu et al., 2017). Similarly, Lei et al. RES exerted chondroprotective effects by activating SIRT1 to inhibit NF-kB activity, thus suppressing NO production in chondrocytes (Lei et al., 2012). In a rabbit model of KOA induced by destabilizing medial meniscus (DMM) surgery, Zhou et al. found that RES supplementation for 2 weeks significantly upregulated SIRT1 activity and thereby inhibited the p53 pathway for chondroprotective effects (Zhou Z. et al., 2021). In addition to modulating the SIRT1-mediated signaling pathway, RES can also modulate multiple other signaling pathways that mediate apoptosis to exert anti-apoptotic effects. *In vitro* KOA model, Liu et al. found that RES reduced chondrocyte apoptosis by activating the PI3K/Akt signaling pathway (Liu and Lv, 2019). Similarly, Jin et al. found that RES protected ATDC5 cells from IL-1β damage by upregulating miR-146b, which inhibited p38 MAPK and NF-kB signaling pathways (Jin et al., 2018). Similarly, Shakibaei et al. found that the anti-apoptotic effect of RES was achieved through activation of the MAPK pathway (Shakibaei et al., 2011). Besides, in primary human chondrocytes treated with RES, Caski et al. observed that RES reduced chondrocyte apoptosis by inhibiting p53 non-ubiquitination-dependent degradation (Csaki et al., 2008). *In vivo* KOA model, Zhang et al. found that RES supplementation may exert anti-apoptotic effects through a DMM-induced KOA mouse model by decreasing metastasis-associated lung adenocarcinoma transcript 1 (MALAT1), NF-kB, and caspase-3. Further *in vitro* studies demonstrated that RES treats KOA by modulating the MALAT1/miR-9/NF-kB axis (Zhang et al., 2020). Furthermore, in an MIA-induced KOA rat model, Wei et al. found that RES exerts anti- KOA effects by activating the heme oxygenase 1/nuclear factor erythroid 2-related factor 2 (HO-1/Nrf2) signaling pathway (Wei et al., 2018). In conclusion, the above-mentioned *in vitro* and animal studies confirmed that RES prevents cartilage loss by promoting chondrocyte proliferation and inhibiting their apoptosis and explored the mechanisms involved in the anti-apoptotic effect. Thus, these studies suggest that RES may alleviate KOA through an anti-apoptotic effect.

### RES and its Role in Maintaining Cartilage Homeostasis in KOA

Cartilage homeostasis is sustained by a dynamic balance between the anabolism and catabolism of cartilage. In the progression of KOA, catabolism dominates and disrupts this dynamic balance, resulting in the loss and degradation of a large amount of cartilage extracellular matrix, ultimately leading to cartilage destruction (Mobasheri et al., 2017). Disruption of cartilage homeostasis, marked by increased production of matrix metalloproteinases (MMPs) and a disintegrin and metalloproteinase with thrombospondin motifs (ADAMTs) and decreased type II (COL-Ⅱ) collagen and aggrecan (AGG), is one of the key factors in the pathogenesis of KOA (Murphy et al., 2002; Kevorkian et al., 2004; Mobasheri et al., 2017). Studies show that RES can play a chondroprotective role by upregulating the chondrocyte anabolic response. Several studies indicate that *in vitro* IL-1β-induced KOA model, RES and its dimer AmurensinH upregulate the anabolic response of cartilage by increasing the expression of COL-Ⅱ and AGG, with a possible mechanism mediated partly through the cartilage-specific transcription factor 9 (SOX-9) (Csaki et al., 2009; Frischholz et al., 2020; Ma et al., 2020; Yi et al., 2020). Similarly, RES plays a chondroprotective role by promoting COL-II expression in KOA chondrocytes (Maepa et al., 2016; Su et al., 2021).

Moreover, RES can also alleviate KOA by mediating the catabolic response of chondrocytes. Studies suggest that RES can improve chondrocyte status *in vitro* KOA model by regulating IL-1β-mediated catabolism. Dave et al. found that RES protected chondrocytes from IL-1β-induced catabolic effects by a mechanism attributed to the reversal of KOA chondrocyte catabolism by RES in part through inhibition of COX-2 expression (Dave et al., 2008). Similarly, Frischholz et al. found that RES culture antagonized IL-1β-induced extracellular matrix degradation and partially rescued IL-1β-injured cartilage matrix deposition in three-dimensional (3D) long-term inflammatory cultures made from porcine chondrocytes in pellet form (Frischholz et al., 2020). Besides, *in vivo* KOA model, RES was found to inhibit chondrocyte metabolic responses, thereby improving chondrocyte function. In a rabbit KOA model established by anterior cruciate ligament transection (ACLT), Elmali et al. found that RES protected rabbit articular cartilage from damage by preventing the loss of proteoglycans from the cartilage matrix (Elmali et al., 2005). Moreover, in the HFD-induced KOA mouse model, Gu et al. found elevated serum levels of C-telopeptide of type Ⅱ collagen (CTX-II) in KOA mice, which is thought to be a degradation product of COL-Ⅱ and could reflect the extent of articular cartilage injury in early KOA. Oral administration of RES in part suppressed KOA cartilage damage by reducing the decomposition of COL-Ⅱ and inhibiting the release of CTX-II(Gu et al., 2016). Additionally, some studies have shown that the accumulation of AGEs in the joint causes cartilage matrix degradation through the upregulation of multiple catabolic products, leading to the loss of glycosaminoglycans (GAG) and collagen (Chen et al., 2002; Yammani et al., 2006; Nah et al., 2008; Rasheed et al., 2011). Several studies suggest that RES exerts a chondroprotective effect by significantly suppressing AGEs-induced MMPs and expression and loss of GAG through activator protein-1 (AP-1) and NF-kB signaling pathways (Liu et al., 2010; Mehta et al., 2021).

Furthermore, NF-kB and TLR4 are considered to be one of the main signaling pathways involved in cartilage catabolism (Sun Y. et al., 2020). Studies have shown that RES maintains endochondral homeostasis by inhibiting the signaling mentioned above pathways. For example, synergistic treatment of RES and curcumin exerted chondroprotective effects by inhibiting the NF-kB pathway (Csaki et al., 2009). Similarly, RES alone showed chondroprotective effects, with the possible mechanism being the downregulation of catabolic factor production through inhibition of the NF-kB pathway (Kang et al., 2018; Yi et al., 2020). Similarly, in an IL-1β-induced human articular cartilage model of KOA, Gu et al. found that RES played a chondroprotective role by inhibiting the TLR4/MyD88 signaling pathway (Gu et al., 2017). It was also found that RES dimer Amurensin H exerts a chondroprotective effect by preventing cartilage matrix loss and cartilage damage through the TLR4/Syk/NF-kB signaling pathway (Ma et al., 2020). Studies have also shown that RES is considered a SIRT1 activator and may act as a chondroprotective agent through the upregulation of multiple SIRT1-mediated signaling pathways (Villalba and Alcaín, 2012). *In vitro* KOA model, Liu et al. found that RES prevented cartilage extracellular matrix degradation by upregulating the SIRT1-mediated Wnt/β-catenin signaling pathway (Liu et al., 2017). Further studies found that intra-articular injection of RES inhibited NF-kB/hypoxia-inducible factor-2α (HIF-2α) axis-induced chondrocyte metabolic responses through activation of SIRT1 in the DMM-induced KOA mouse model, thus exerting a chondroprotective effect (Li et al., 2015). However, inconsistent with previous studies, Kim et al. processed KOA chondrocytes with RES *in vitro* and observed that RES significantly upregulated the expression of SIRT1, runt-related transcription factor 2 (RUNX2), collagen type Ⅰ protein (COL-Ⅰ), and collagen type Ⅹ protein (COL-Ⅹ) in chondrocytes to induce chondrocytes into a hypertrophic state (Kim et al., 2014). Similarly, Eo et al. found that RES extract (IS526) induced cartilage destruction via activation of Erk1/2 and PI3K pathways in rabbit chondrocytes (Eo S.-H. and Kim S. J., 2020). These contrary findings to expectations suggest that we should consider whether different modes of administration, different concentrations of RES or, different ways of extracting RES may have opposite pharmacological effects on chondrocytes. In conclusion, RES, as an active ingredient in natural medicine with chondroprotective effects, may hold great promise in improving KOA by maintaining cartilage homeostasis.

### RES and its Role in Promoting Autophagy in KOA

Autophagy is a physiological process characterized by cells transferring their complexes into lysosomes for degradation by combining their own abnormal or senescent cytoplasmic proteins or organelles into autophagic vesicles. Autophagy performs a crucial role in maintaining intracellular energy metabolism and regulating nutrition (Li et al., 2016). Studies show that as cells age, the autophagic activity decreases, and various intracellular macromolecular proteins accumulate and cannot be removed, eventually leading to cell degeneration and apoptosis (Tsujimoto and Shimizu, 2005). Recently, with the increasing understanding that activation of autophagy can negatively regulate chondrocyte apoptosis, the study of autophagy-related pathways and their regulators in the progression of KOA has received extensive attention from researchers (Schwartz et al., 1993). Among them, the mammalian target of rapamycin (mTOR) (autophagy-related negative regulator) upregulates and inhibits the autophagic activity of chondrocytes in KOA to cause apoptosis of many chondrocytes, which eventually leads to apoptosis cartilage degeneration (Vasheghani et al., 2015; Sun K. et al., 2020). The above suggests that promoting autophagic activity may be a novel therapeutic approach for anti- KOA.

Studies suggest that *in vivo* model, RES exerts chondroprotective effects by upregulating autophagy-related pathways. For example, in a DMM-induced KOA mouse model, Qim et al. found that RES promoted cartilage autophagy and slowed articular cartilage degeneration in KOA mice by upregulating the expression of hypoxia factor 1α (HIF-1α) and HIF-2α through the AMP-activated protein kinase (AMPK)/mTOR signaling pathway (Qin et al., 2017). However, studies addressing the straightforward actions of RES on KOA -promoted autophagy are limited, and future studies are still needed to confirm the chondroprotective role of RES in KOA. Therefore, the above studies suggest that it is still possible for RES to mitigate KOA by mediating autophagy to improve the function of KOA chondrocytes.

## Clinical Studies About RES and its Effects in KOA

RES is one of the most common bioactive supplements from natural plants and has been used with favorable results in various chronic diseases (Bo et al., 2013; Chen et al., 2019; Galiniak et al., 2019). In *vitro* experiments and animal models of KOA, RES showed chondroprotective effects, potentially owing to a reduction in the production of inflammatory factors and apoptotic molecules. However, there are still few studies on RES in KOA patients, but all have shown good efficacy.

Pain and joint stiffness are among the problems that plague most people with KOA, and reducing pain and restoring mobility is considered the main goals of KOA treatment (Min et al., 2017). Studies have shown that RES supplementation significantly relieves pain and improves joint stiffness in patients with KOA. In a randomized, double-blind study of 110 subjects over 3 months, RES (500 mg/d) combined with meloxicam (MIx) (15 mg/d) was found to significantly improve pain and enhance joint function in patients with KOA compared to the placebo group after 3 months of treatment, and clinical and biochemical indices indicated that RES was safe and tolerated as an adjunct to MIx in patients with KOA (Hussain et al., 2018). In addition, serum inflammatory factor levels were significantly reduced in KOA patients after 90 days of RES treatment, consistent with the conclusion in animal model studies that RES has anti-inflammatory effects (Marouf et al., 2018). In a further study, Marouf et al. found that oral RES (500 mg/day) for 90 days significantly improved patients’ knee pain and mobility but had no significant effect on patients’ serum Col-II levels. However, due to the limitations of the trial, such as short intervention time, small sample size, and no control group, further studies are needed to confirm the results in the future (Marouf, 2021). Furthermore, pain is one of the common complaints of postmenopausal women. Pain due to vascular dysfunction caused by decreased estrogen levels in postmenopausal women plays a key role in the development and progression of age-related KOA (Laskarin et al., 2016). Studies suggest that RES supplementation may improve chronic pain in postmenopausal women. In several randomized, double-blind studies of healthy postmenopausal women, RES supplementation (75 mg twice daily) reduced chronic pain in age-related arthritis, alleviated somatic symptoms of menopause, and improved the quality of life associated with postmenopausal women compared to placebo treatment (Wong et al., 2017; Thaung Zaw et al., 2020). In addition to pain and joint stiffness, the local inflammatory response in patients with KOA is challenging in treatment. In another study, the authors et al. looked at the correlation between serum pro-inflammatory cytokines and clinical scores in 110 patients with KOA eligible for RES (500 mg/d) combined with Mix (15 mg/d) treatment. The study results showed that RES combined with MIx treatment reduced serum inflammatory biomarkers in patients with KOA. However, no significant correlation between serum biomarkers and clinical outcomes has been found, and substantial future research on their correlation is still needed (Marouf et al., 2021). In conclusion, RES has shown significant effects on improving symptoms such as pain and joint stiffness in patients with KOA. However, future studies are still needed to confirm the specific mechanism of action of RES in patients with KOA.

## New Applications of RES in KOA

**FIGURE 3:**
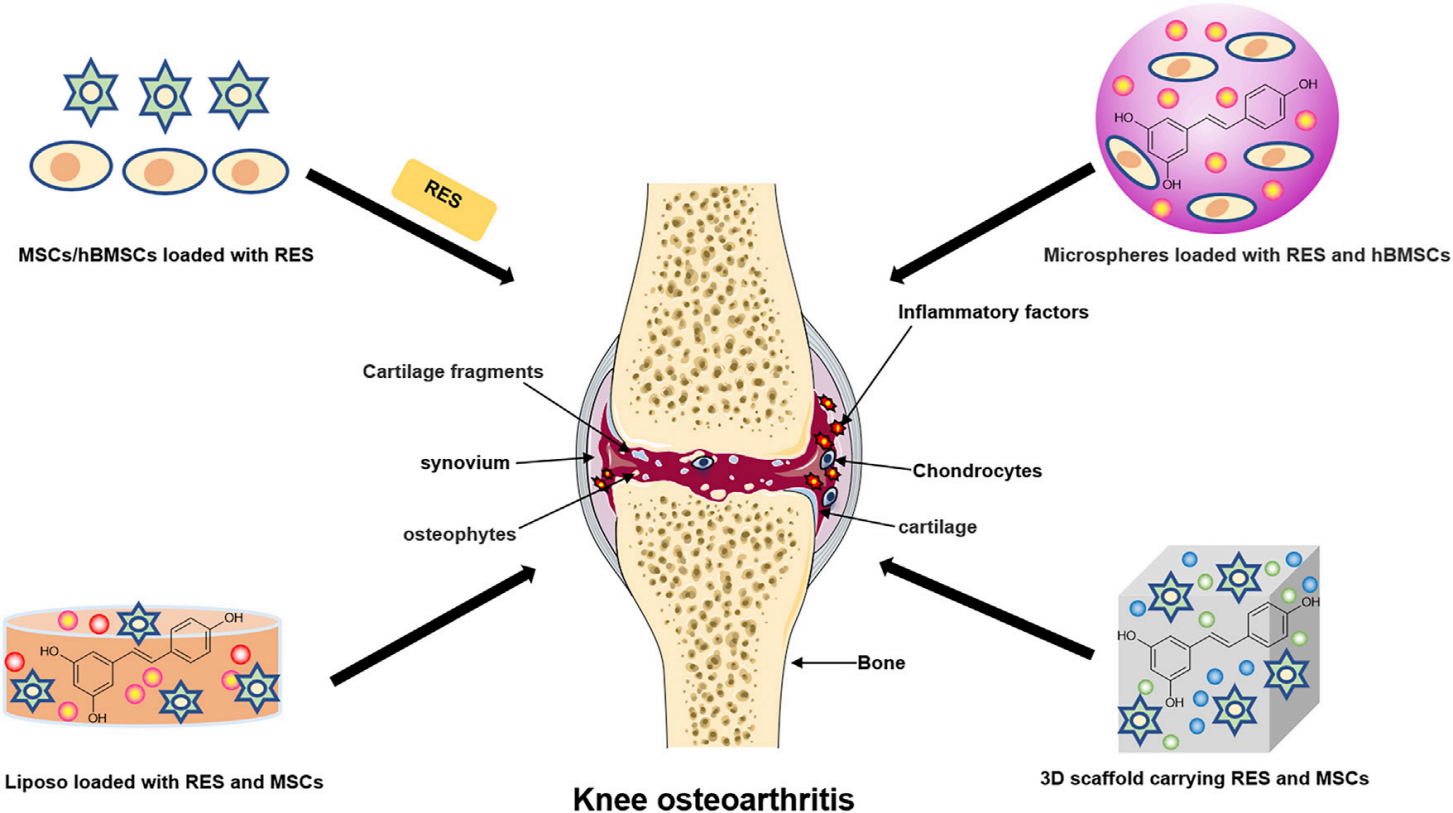
The new application of resveratrol in knee osteoarthritis. The schematic diagram illustrates the current clinical method of combining resveratrol with cartilage tissue engineering to treat knee osteoarthritis.

Destruction of articular cartilage or degeneration of cartilage due to other pathological factors is the leading cause of the development of KOA, which can eventually lead to progressive and complete joint destruction (Charlier et al., 2019). It is worth mentioning that studies have shown that the development of synovial inflammation is associated with cartilage damage. The inflammatory mediators released during synovial inflammation activate inflammatory pathways, thereby damaging cartilage (Sanchez-Lopez et al., 2022). Current treatment of KOA focuses on inflammation, pain and improvement of joint function, and some conventional drugs such as NSAIDs and opioids are less effective and have potential side effects in treating KOA. In addition, researchers have proposed a new class of drugs called Disease-Modifying Therapies for Osteoarthritis (DMOADs) to treat KOA by blocking structural changes in the joints and improving symptoms. However, such a drug has not yet been developed (Oo et al., 2021). Therefore, there is a need to find alternative and better regeneration methods. Tissue engineering (TE) is a promising field that combines biological sciences and engineering to create potential biologically based alternatives to repair remodel damaged tissue function. The TE strategy opens up new breakthroughs and prevents progress in KOA when articular cartilage is damaged and cannot be regenerated and repaired (Griffith and Naughton, 2002; Deng et al., 2003). Besides, natural, nontoxic herbs from nature are extensively used to treat many diseases due to their multi-targeted and other biological activities (Buhrmann et al., 2020). In fact, research on the appropriate use of natural compounds in TE should be considered a worthwhile approach for treating KOA. (Figure 3 and Table 2).

**TABLE 2:** New applications of RES in OA.

Main effects	Study type	Animals/Cells	Experimental model	References
-Enhanced MSCs of the chondrogenic differentiation potential	*In vitro* and *vivo* study	MSCs Osteochondral defect New Zealand white rabbit model	Hy/MSC with resveratrol	Choi et al. (2018)
-Increased cartilage regeneration potential				
-Increased ECM proteins, COL-Ⅱ, and AGG levels				
-Decreased COL-Ⅹ levels				
-Suppressed NF-kB signaling pathway	*In vitro* study	IL-1β-induced MSCs	MSC-derived chondrocyte morphology cultured on CGS	Lei et al. (2008)
-Inhibited MMP-13 levels				
-Increased aggrecan and COL-Ⅱ levels				
-Increased resveratrol bioavailability	*In vitro* study	hBMSCs	RES-loaded PLGA microsphere	Wu et al. (2016)
-Increased SOX-9, COL-Ⅱ, AGG, and MMP-13 levels				
-Inhibited MAPK and NF-kB signaling pathway	*In vitro* study	Human chondrocytes and chondrosarcoma	In 3D-alginate cultures and treated with resveratrol	Im et al. (2012)
-Inhibited MMP-13, ADAMTS4, and ADAMTS5 levels				
-Suppressed p53-induced apoptosis				
-Enhanced chondrogenic differentiation and proliferation of hWJSCs	*In vitro* study	hWJSCs	Resveratrol incubation in HG	Sultan et al. (2020)
-Increased COL-Ⅱ and SOX-9 levels				
-Decreased COL-Ⅰ and COL-Ⅹ levels				
-Decreased IL-1α, IL-6, and IL-8 levels				
-Increased G-CSF and GM-CSF levels				
-Decreased NO levels	*In vitro* study	Human chondrocytes	Lipid-core nanocapsules with resveratrol and curcumin	Kann et al. (2016)
-Lower ROS and H_2_O_2_ levels	*In vitro* study	Rabbit chondrocytes	Lip-RES and RES	Quan et al. (2017)
-Suppressed PI3K/Akt signaling pathways	*In vitro* and *vivo* study	Chondrocytes Osteochondral defect SD rat model	Resveratrol–PLA–gelatin porous nano-scaffold	Yu et al. (2018)
-Increased SIRT1 expression				
-Decreased VEGF, PTEN, caspase-9, and MMP-13 levels				
-Increased COL-Ⅱ levels				
-Promoted cartilage repair	*In vitro* study	Chondrocytes Osteochondral defect SD rat model	Resveratrol-loading PLA/Gelatine 3D nano-scaffolds	Ming et al. (2018)
-Increased AGG, COL-Ⅱ, and SOX-9 levels	*In vitro* study	Mice chondrocytes	Oxi-HA/RES	Sheu et al. (2013)
-Decreased IL-1β, MMP-1, MMP-3, and MMP-13 levels				
-Decreased TNF-α and IL-1β levels	*In vitro and vivo* study	Rat chondrocytes ACLT-induced OA rat model	(RES/CellROX@ZIF-8) NPs	Zhang et al. (2021)
-Lowered ROS levels				
-Suppressed iNOS expressions				
-Inhibited oxidative stress	*In vitro* study	T/C28a2	Resveratrol self-emulsifying systems	Le Clanche et al. (2018)

Abbreviations: **RES**, resveratrol; **KOA**, knee osteoarthritis; **MSCs**, mesenchymal stem cells; **ECM**, extracellular matrix; **COL-Ⅱ**, type II collagen; **AGG**, aggregate; **COL-Ⅹ**, collagen type Ⅹ protein; **IL-1β**, interleukin 1β; **CGS,** chitosan-gelatin scaffolds; **NF-kB**, nuclear factor-kappa B; **MMP-13**, matrix metalloproteinase 13; **hBMSCs**, human bone mesenchymal stem cells; **PLGA**, Poly(lactic-co-glycotic acid); **SOX-9**, cartilage-specific transcription factor 9; **3D**, three-dimensional; **MAPK,** mitogen activated protein kinase; **ADAMTs-4**, a disintegrin and metalloproteinase with thrombospondin motifs-4; **ADAMTs-5**, a disintegrin and metalloproteinase with thrombospondin motifs-5; **hWJSC,** human Wharton’s gel stem cell; **HG,** high glycemic**; COL-Ⅰ**, collagen type Ⅰ protein; **IL-6**, interleukin 6; **IL-8**, interleukin 8; **G-CSF**, granulocyte-colony stimulating factor; **GM-CSF**, granulocyte-macrophage colony-stimulating factor; **NO**, nitric oxide; **ROS**, reactive oxygen species; **H**_**2**_ **O**_**2**_, hydrogen peroxide; **SD**, Sprague Dawley; **PI3K**, phosphatidylinositol-3-kinase; **Akt**, protein kinase B; **SIRT1**, sirtuin1; **VEGF**, vascular endothelial growth factor; caspase-9, cysteine aspartate protease-9; **Oxi-HA/RES**, RES-containing hyaluronic acid/hydrogel; **AGG**, aggregate; **MMP-1**, matrix metalloproteinase 1; **MMP-3**, matrix metalloproteinase 3; **TNF-α**, tumor necrosis factor-α; **ACLT**, anterior cruciate ligament transection; **RES@ZIF-8-MPEG-TK**, methoxy polyethylene glycol-solidated RES and CellROX zeolite-based imidazole salt backbone-8 nanoparticles; **ROS**, reactive oxygen species; **iNOS**, nitric oxide synthase; **T/C28a2**, human immortalized chondrocyte cell line.

RES is considered a potential target for application in cartilage TE for KOA treatment due to its proven good cartilage protective effects. Currently, several studies have investigated the effects of RES combined with TE on cartilage. Mesenchymal stem cells (MSCs), due to their ability to differentiate and regenerate cartilage, are considered complementary and alternative sources of chondrocytes (Uccelli et al., 2008). MSCs have been shown to bind well to cartilage biomaterials and bioactive factors and are increasingly used in KOA treatment. Studies have shown that RES treatment significantly promotes the differentiation of MSCs to chondrocytes (Choi et al., 2018). In addition, Lei et al. found that co-treatment of RES with transforming growth factor 3 (IGF-3) promoted the differentiation of MSCs attached to chitosan-gelatin scaffolds (CGS) toward chondrocytes antagonized the chondrocyte catabolic response induced by IL-1β stimulation by inhibiting the NF-kB signaling pathway (Lei et al., 2008). Similarly, Wu et al. developed RES slow-release microspheres that significantly improved the bioavailability of RES and promoted human bone mesenchymal stem cells (hBMSCS) differentiation to chondrocytes, and inhibited IL-1β-induced ground MMP-13 expression, which were considered as potential approaches to promote cartilage repair and KOA therapy (Wu et al., 2016). In chondrocytes cultured with 3D alginate beads, Im et al. found that RES significantly increased the synthesis of bone morphogenetic protein 7 (BMP7) and inhibited signaling pathways involved in KOA inflammatory and chondrogenic metabolic effects such as MAPK and NF-kB (Im et al., 2012). Moreover, Sultan et al. found that RES can stimulate human Wharton’s gel stem cell (hWJSC) differentiation towards chondrocytes by regulating the levels of inflammatory factors *in vitro* in a high glycemic (HG) state (Sultan et al., 2020).

In recent years, numerous studies have shown that 3D nano-scaffold materials are widely used for cartilage repair because of their excellent robustness, biodegradability, and biocompatibility (Kim et al., 2001; Elzoghby et al., 2012). The combination of RES and nanomaterials for KOA treatment has received increasing attention. For example, Kann et al. designed lipid-core nanocapsules (LNC) containing RES and curcumin, and LNC pretreatment significantly inhibited SNP-induced NO expression and subsequent cartilage apoptosis in human chondrocytes. Moreover, polyphenol-loaded nanocapsules treated with chondrocytes were protective of cell morphology and membrane surface as observed by atomic force microscopy (AFM), indicating a chondroprotective effect of TE (Kann et al., 2016). In a similar study, Quan et al. examined the inhibitory effect of RES-loaded nanoliposomes (Lip-RES) and free RES on SNP-induced apoptosis in rabbit chondrocytes. However, contrary to expectations, free RES better-reduced chondrocyte apoptosis, which may be caused by the slow release of RES from it due to the lipophilic nature of Lip-RES (Quan et al., 2017). Similarly, studies reported that after implanting polylactic acid-gelatin nano scaffolds containing RES into cartilage-deficient rats, good recovery was observed at the site of cartilage damage in rats, possibly through the activation of the PI3K/Akt signaling pathway (Ming et al., 2018; Yu et al., 2018). Furthermore, Sheu et al. found that hyaluronic acid/hydrogel containing RES (Oxi-HA/RES) could be well incorporated into chondrocytes to replace the lost cartilage matrix due to its good biocompatibility and reduce lipopolysaccharide (LPS)-induced inflammatory damage. The above suggests that Oxi-HA/RES may be a potential cellular vehicle for TE treatment of cartilage defects (Sheu et al., 2013). Notably, Zhang et al. developed a novel nanodevice (RES/CellROX@ZIF-8-mPEG-TK) containing RES, which can detect the level of ROS in KOA chondrocytes. Besides, the device can promote the conversion of macrophages from MA1 (pro-inflammatory) to M2 (anti-inflammatory), thereby reducing cartilage degeneration *in vivo* and achieving integrated KOA inflammation treatment (Zhang et al., 2021). Besides, the RES nanoemulsion system designed by Clanche et al. significantly increased the tolerance of human immortalized chondrocyte cell line (T/C28a2) to RES and protected oxidative stress-mediated cell death T/C28a2 (Le Clanche et al., 2018). In summary, RES has potential biotherapeutic effects in preventing and treating degenerative cartilage diseases and can be used as a unique biotherapeutic tool in combination with TE for better prevention and treatment of KOA.

## Summaries and Perspectives

In summary, RES, as a natural compound, may play a relevant role in preventing and treating KOA. Preclinical trials and clinical trial studies have suggested that RES has anti-inflammation, anti-apoptosis, cartilage homeostasis maintaining, and autophagy promoting effects. RES exerts a positive effect in reducing inflammatory activation, apoptosis, and cartilage degeneration in KOA and is considered to show a potential beneficial action against KOA. RES is considered a safe and effective drug with few side effects and is expected to be an alternative therapy for prevention and treatment.

Although the research on RES’s anti-osteoarthritic activity has witnessed significant advances in recent years, it still has many unanswered questions. The first is that, while a great deal of research has confirmed the effectiveness of RES in KOA treatment, the specific mechanisms and potential molecular targets of RES on KOA still need to be further explored. Moreover, in the second place, the studies on the pharmacological effects of RES on subchondral bone and synovium are still limited, even though it is substantiated that RES shows promising therapeutic potential for KOA chondrocytes. Besides, current research on KOA is confined to preclinical studies. Evidence from *in vitro* studies suggests that RES offers a promising natural therapeutic agent for treating and preventing KOA by acting on chondrocytes. However, since *in vitro* cell culture systems do not consider bioavailability, further animal studies are needed to complement the preclinical evidence for RES prevention of KOA. In addition, clinical trials addressing the efficacy of RES in KOA patients deserve further investigation. It is feasible to modify the molecular structure of RES or combine it with other compounds to obtain multi-targeted RES derivatives because of its low bioavailability. That is to say, and future research is needed to focus on the synthesis of RES derivatives to improve bioavailability and enhance the curative effect of KOA. Furthermore, although numerous *in vitro* and animal experiments provide data references for clinical trials, the potential molecular mechanisms of RES in KOA are not yet clear. Moreover, the pharmacokinetics and toxicities of RES in KOA still need to be further explored through extensive clinical trials. Finally, RES has been shown to have satisfactory efficacy as an antioxidant in various diseases. However, studies on the anti-oxidative stress of RES in KOA are still very limited. In the future, further studies are needed to confirm the therapeutic role of RES as an antioxidant in KOA.

To sum up, more preclinical studies and clinical trials are still required to verify the efficacy of RES and to elucidate the various mechanisms by which RES may alleviate KOA, which may provide clinical implications for intervention in the development of KOA.
